# Current News

**Published:** 1894-07

**Authors:** 


### Current News.

### AMERICAN DENTAL ASSOCIATION.

THE Thirty-fourth Annual Session of the American Dental Association will be held at Old Point Comfort, commencing at 10 A.M., Tuesday, August 7, 1894.

GEO. H. CUSHING,
*Recording Secretary.*

### AMERICAN DENTAL SOCIETY OF EUROPE.

THE American Dental Society of Europe will hold its nineteenth meeting at Geneva, Switzerland, August 6, 7, and 8, 1894. Last year this annual gathering was omitted, as many members wished to attend the Columbian Dental Congress. American colleagues who may be taking a summer vacation abroad are cordially urged to combine with their plans for travel and sight-seeing a visit to their professional brethren in Europe, and to join in the various exercises of the occasion. Members of the profession everywhere will be welcome. Programmes may be had on application to the President, Dr. L. C. Bryan, of Basel, or to the undersigned.

CHARLES W. JENKINS,
*Secretary.*

NO. 1 SONNENQUAI, ZURICH.

---

### SPECIAL NOTICE.—MEETING OF THE AMERICAN DENTAL ASSOCIATION AT OLD POINT COMFORT, AUGUST 7.

ARRANGEMENTS are under way, but have not yet been completed, for a reduction in railroad fare for the annual meeting of the American Dental Association, to be held at Old Point Comfort in August next.

We expect a reduction of one and one-third fare for the round trip on the "Certificate Plan." In order to get this reduction, full fare must be paid in going to the meeting, *and a receipt taken therefor from the ticket-agent at the starting-point* This receipt (certificate) must be countersigned by the Secretary of the Association at the meeting, and will enable the holder to return for one-third fare, provided one hundred certificates are presented, showing that at least that number have paid full fare on the various railroad lines entering the place where the meeting is held.

A circular giving full particulars will be mailed later to all members and to those who apply for information.

J. N. CROUSE,
*Chairman.*

2231 PRAIRIE AVENUE, CHICAGO, June 18, 1894.

---

### ILLINOIS STATE DENTAL SOCIETY.

AT the Thirtieth Annual Meeting of the Illinois State Dental Society, held at Springfield, May 8–11, 1894, the following officers were elected for the ensuing year:

J. W. Cormany, Mount Carroll, President; S. F. Duncan, Joliet, Vice-President; Louis Ottofy, Chicago, Secretary; W. A. Stevens, Chicago, Treasurer; Grafton Munroe, Springfield, Librarian.

The next meeting will be held at Galesburg May 14–17, 1895.

LOUIS OTTOFY,
*Secretary.*

MASONIC TEMPLE, CHICAGO.

